# 5-years follow-up of patients with the clinical high-risk state for psychosi

**DOI:** 10.1192/j.eurpsy.2021.2005

**Published:** 2021-08-13

**Authors:** V. Kaleda, M. Omelchenko

**Affiliations:** 1 Department Of Youth Psychiatry, FSBSI «Mental Health Research Centre», Moscow, Russian Federation; 2 Department Of Youth Psychiatry, FSBSI Mental Health Research Center, Moscow, Russian Federation

**Keywords:** high risk psychosis, early intervention, youth depression, attenuated positive symptoms

## Abstract

**Introduction:**

The identification of the psychosis high-risk state in help-seeking patients with depressive symptoms offers the possibility of detection and intervention at the early stages of schizophrenia.

**Objectives:**

Estimating the 5-year follow-up rate of the manifestation of psychosis and levels of functioning in patients with the clinical high-risk state and depressive symptoms.

**Methods:**

81 inpatients (average age 19.6 years) with depressive symptoms and attenuated psychosis (60 patients with APS and 21 patients with BLIPS). Average duration of inpatient treatment was 56.3 days, antidepressant therapy (mean dosage equivalent to fluoxetine 43.1 mg/day) and antipsychotic therapy (mean dosage equivalent to chlorpromazine 408.9 mg/day) were conducted. All patients were followed up after discharge at least during 5 years (average follow-up 7.1 years). Levels of functioning were assessed on the PSP scale.

**Results:**

The manifestation of psychosis was identified in 21.0% (17 patients) (on average in the third year of follow-up), complete symptomatic and functional remission was established in 11.1% (9 patients) (PSP 100-81), complete symptomatic and incomplete functional remission was established in 27.2% (22 patients) (PSP 80-61). Incomplete symptomatic and incomplete functional remission – in 24.7% (20 patients) (PSP 60-41) and 13.5% (11 patients) (PSP<40).

**Conclusions:**

The combination of antidepressants and antipsychotics therapy in patients with the clinical high-risk state for psychosis reduced the risk of psychosis manifestation but did not significantly affect the level of outcome compared to other studies.

**Disclosure:**

No significant relationships.

